# Tree biomass in the Swiss landscape: nationwide modelling for improved accounting for forest and non-forest trees

**DOI:** 10.1007/s10661-017-5816-7

**Published:** 2017-02-15

**Authors:** B. Price, A. Gomez, L. Mathys, O. Gardi, A. Schellenberger, C. Ginzler, E. Thürig

**Affiliations:** 1 Swiss Federal Institute for Forest, Snow and Landscape Research (WSL), Zürcherstrasse 111, 8903 Birmensdorf, Switzerland; 2 Nategra LLC, Nydeggstalden 30, 3011 Bern, Switzerland; 3 Sigmaplan AG, Thunstrasse 91, 3006 Bern, Switzerland; 4 School for Agricultural, Forest and Food Sciences, Bern University of Applied Sciences HAFL, Länggasse 85, 3052 Zollikofen, Switzerland; 5 Climate Division, Federal Office for the Environment FOEN, 3003 Bern, Switzerland

**Keywords:** Tree biomass, ALS, Image point clouds, Aerial imagery, Trees outside forest (TOF)

## Abstract

Trees outside forest (TOF) can perform a variety of social, economic and ecological functions including carbon sequestration. However, detailed quantification of tree biomass is usually limited to forest areas. Taking advantage of structural information available from stereo aerial imagery and airborne laser scanning (ALS), this research models tree biomass using national forest inventory data and linear least-square regression and applies the model both inside and outside of forest to create a nationwide model for tree biomass (above ground and below ground). Validation of the tree biomass model against TOF data within settlement areas shows relatively low model performance (*R* ^2^ of 0.44) but still a considerable improvement on current biomass estimates used for greenhouse gas inventory and carbon accounting. We demonstrate an efficient and easily implementable approach to modelling tree biomass across a large heterogeneous nationwide area. The model offers significant opportunity for improved estimates on land use combination categories (CC) where tree biomass has either not been included or only roughly estimated until now. The ALS biomass model also offers the advantage of providing greater spatial resolution and greater within CC spatial variability compared to the current nationwide estimates.

**Electronic supplementary material:**

The online version of this article (doi:10.1007/s10661-017-5816-7) contains supplementary material, which is available to authorized users.

## Introduction

Within the United Nations framework convention on climate change (UNFCCC), 197 parties are currently committed to reporting their annual greenhouse gas emissions. Switzerland ratified the UNFCCC on 10 December 1993 and its Kyoto Protocol on 9 July 2003. In addition, Switzerland has ratified the Doha amendment for the second commitment period of the Kyoto protocol (2013–2020) and targets a 50% reduction in greenhouse gas emissions relative to 1990 levels by 2030 (Swiss Confederation 2015). In this context, reporting on emissions and removals of greenhouse gas from land use, land-use change and forestry (LULUCF) is required and as such biomass status and fluxes have to be estimated, as well as carbon accounting for forest management (FOEN 2016a). The *2006 IPCC Guidelines for National Greenhouse Gas Inventories* (IPCC 2006), volume 4, Agriculture, Forestry and Other Land Use, defines six key land use categories (forest land, cropland, grassland, wetlands, settlements, other land) to which the greenhouse gas inventory (GHGI) reporting applies.

Forest inventories have a long tradition in quantifying above ground wood volumes representative over large areas, mostly related to timber production (Tompo et al. 2010). Many forest inventories quantify below ground biomass and above ground biomass (AGB) as timber volume based on a large range of different methods (Mohren et al. 2012; Neumann et al. 2016). Methods used are mostly based on field measurements at the tree level that are then related to measured timber volume from felled trees. Frequently used field measurements include diameter at breast height (DBH), tree heights at distinct levels of the tree and tree type. These measurements are then related to measured timber volume through expansion factors (Petersson et al. 2012) or allometric functions (Chave et al. 2005, 2014; Zianis and Mencuccini 2004). Assuming parameterisation of allometric functions or expansion factors have no bias, tree biomass can be well estimated by that data.

Often, a significant proportion of total tree cover is found outside forests. All trees that occur outside of areas officially defined as forest or other wooded land are defined by the UN’s Food and Agriculture organisation as trees outside forest (TOF) (FAO 2001). TOF can perform a variety of social, economic and ecological functions including carbon sequestration (Atangana et al. 2014; Idol et al. 2011; Schnell et al. 2015). Detailed quantification of biomass for trees, however, is usually limited to forest areas, partly because of these areas having the greatest relevance as carbon pools but also because allometric functions or expansion factors for trees are parameterised with trees from forest areas, where inventory data is available. Therefore, there is potential need for national-level information on TOF from stakeholders such as landowners and national governments (Schnell et al. 2015). While numerous studies have investigated quantifying tree biomass on other land use types such as settlements (e.g. Nowak and Crane 2002; Hutyra et al. 2011; Strohbach and Haase 2012; McPherson et al. 2013; Gardi et al. 2016) and cropland (e.g. Huffman et al. 2015; Zhou et al. 2015), recent studies have found that consistent and systematic assessment of TOF at the national level is largely lacking (Schnell et al. 2015, but see Guo et al. 2014). Substantial proportions of land based carbon storage have been shown to be found on non-‘forest’ land use types (where definition of forest may be important) for example in China (Guo et al. 2014) and the USA (Churkina et al. 2010). Across Switzerland, over 6% of trees are located outside forest areas (Ginzler et al. 2011). In Switzerland, GHGI biomass reporting for areas outside forest is based on land use categories with biomass estimates based on best available research studies or expert knowledge (FOEN 2016b).

Information on TOF can be inconsistent in its coverage on different land use types and fragmented spatially or temporally (Schnell et al. 2015). These inconsistencies can result from variations in jurisdiction for land management agencies or individuals. In particular, access to private land for field measurement can prove challenging (Schnell et al. 2015), and as such, models based on remotely sensed data offer many advantages.

Models to quantify tree biomass across non-forest areas can be developed using a variety of methods including using remote sensing data, in particular, taking advantage of structural information available from stereo imagery or airborne laser scanning (ALS)/light detection and ranging (LiDAR; van Leeuwen et al. 2011; Zolkos et al. 2013) or a combination of both (Asner et al. 2010; Gonzalez et al. 2010). The advantages of ALS data over aerial imagery for modelling vegetation structure are largely due to the ability of laser data to penetrate the canopy and as such offer more detailed information on vegetation structure. As reviewed by Wulder et al. (2012), ALS data has been used to support modelling and mapping of forest structure attributes over large areas in numerous cases and through a variety of approaches. However, capture of ALS data can be expensive both in terms of time and monetary measures, and datasets with regular repeats over large areas are rare. As such, ALS data is often incorporated into large area modelling and mapping projects through a sample based approach and sometimes up-scaled using optical satellite imagery (Armston et al. 2009; Asner et al. 2010; Moffiet et al. 2010). While ALS data is available covering almost all of Switzerland, this data is a patchwork of separate projects with varying specifications and capture dates and does not cover high elevation areas above approximately 2000 m asl. On the other hand, aerial stereo imagery is captured wall-to-wall within Switzerland on an ongoing basis with a repeat cycle of 6 years by the Federal Office for Topography (Ginzler and Hobi 2015). Point cloud datasets derived from this data, following stereo matching, also offer the ability to measure canopy structure (but canopy surface only with no point penetration of the canopy) and therefore model tree biomass.

Recent work by Schnell et al. (2015) measures the contribution of TOF to national level biomass stocks in 11 countries over three continents. No European countries are included in the study. We contribute further to the knowledge on the contribution of TOF through a case study in Switzerland and model tree biomass (above ground and below ground) nationwide.

This research aims to create a nationwide model for tree biomass (above ground and below ground), both inside and outside of forest, built on structural information from point cloud data derived from airborne data. In this context, we quantify woody biomass across Switzerland, including trees outside forest. We compare the ability of ALS data to that of aerial stereo imagery to model tree biomass and determine the degree of benefit of canopy penetrating ALS data over the cost-effective and regularly updated aerial imagery data (which provides canopy surface data only). We evaluate estimated tree biomass against validation data for TOF and demonstrate the utility of the modelling approach across a large nationwide heterogeneous area. We compare the tree biomass model with current estimates used for GHGI and carbon accounting.

## Methods

### Study area

Switzerland is a central European country on the Alpine arc with altitudes ranging from 200 m asl to 4600 m asl covering a central plateau, Alpine regions and the rolling mountains of the Jura. The Swiss GHGI reports on the entire country with an area of approximately 42,000 km^2^ (FOEN 2016b). Forests cover 31% of Switzerland and have increased between the last two consecutive forest inventories, national forest inventory (NFI) 2 (1993–1995) and NFI3 (2004–2006), by 4.9% (Brändli 2010). Deciduous trees make up 47.1% and coniferous trees 52.9% of the forest species composition.

### Data

The Swiss national forest inventory is a regularly updated inventory of Swiss forest assets, incorporating aerial imagery interpretation and fieldwork. The first inventory was 1983–1985 (EAFV 1988), NFI2 1993–1995 (Brassel and Brändli 1999), NFI3 2004–2006 (Brändli 2010) and the current inventory (NFI4) is a continuous survey which began in 2009 and runs until 2017 (Abegg et al. 2014). In this research NFI3 and NFI4 data were used. Within the NFI, forest area is determined by aerial photography interpretation and defined as area of cover of trees over 3 m in height at least 50 m wide and with greater than 20% projected crown cover. With greater crown cover, a decrease in width of the treed area is allowed down to a minimum of 25 m at 100% crown cover (Brassel and Lischke 2001). This differs from the Kyoto Protocol definition used by Switzerland where a forest is a treed area wider than 25 m with more than 20% crown cover (FOEN 2016a). Tree biomass is derived from field measurements on the terrestrial sample plots of the NFI (Brassel and Lischke 2001). In case of ambiguity in aerial photo interpretation, determination of forest or non-forest is performed in the field. Plots clearly identified as non-forest from remote interpretation are not visited in the field and have thus no information on tree biomass. The terrestrial sample grid follows a regular distribution of 1.4 km spacing. Sample plots consist of two concentric circles with a size of 200 m^2^ for trees with DBH between 12 and 36 cm and a size of 500 m^2^ for trees with DBH ≥ 36 cm. This results in DBH measurements of approximately 11 trees per plot. On a sub-sample of approximately two trees per plot, diameter at tree height 7 m (d_7_) and tree height (h) are measured. The biomass estimation of all single trees is based on allometric functions. Volume of stem-wood over bark including stock, coarse branches (≥7 cm) and small branches (<7 cm) are based on Brassel and Lischke (2001) converted to biomass by multiplication with species specific basic wood densities of Assmann (1961). The estimation of needles/leaves is based on Perruchoud et al. (1999). Estimates for branches, foliage and roots are derived from DBH only; for stem-wood over bark including stock, the d_7_ and h measurements are also required (Brassel and Lischke 2001).

ALS and airborne stereo imagery (ADS80) are available nationwide from which structural vegetation properties are derived as explanatory variables. ALS data has been flown between 2001 and 2014, through a variety of projects which combined provide wall-to-wall coverage of Switzerland, excluding high elevation areas above approximately 2000 m asl. As such, the specifications vary spatially, but with an average point density of 0.5 points/m^2^. A digital terrain model derived from the ALS data provides topographic data and also allows derivation of the vegetation height data from the ALS data and the aerial imagery. Aerial imagery captured with the ADS40/ADS80 sensors has been captured wall-to-wall across Switzerland by Swisstopo (the Federal Office for Topography) on a continuing cycle since 2007, with repeat coverage every 6 years. To derive the vegetation height model, summer imagery from 2007 to 2012 were used for stereo-matching with ground sample distance of 0.25 m in the central plateau and 0.5 m in mountainous areas (Ginzler and Hobi 2015).

The Disaster Monitoring Constellation (DMC) is a constellation of several satellites which provides spectral imagery in the red, green and near infrared bands at a spatial resolution of 22 m (Crowley 2008). Cloud-free composites covering the whole of Switzerland are available on a monthly basis between 2012 and 2014 from which we derived normalised difference vegetation index (NDVI) data.

Climate variables used in modelling were derived from the baseline Swiss climate dataset of (Zimmermann and Kienast 1999), which related to climate period of 1961–1990.

Validation data was available for areas of TOF through the urban tree inventory of the community of Bern (Gardi et al. 2016). This inventory included 21 felled trees with direct measures of AGB and 179 plots of 25 m × 25 m where tree height and DBH were measured, and above ground biomass modelled using allometric functions of Chave et al. (2014) and treated as observed data (fit to felled trees of *R* ^2^ = 0.96). This data was converted to total tree biomass (above ground and below ground) using the roots to shoots ratio of 0.26 (Nowak and Crane 2002) for comparison to the GHGI and our modelled tree biomass.

Biomass estimates for the GHGI are defined for land use ‘combination categories’ (CCs) which are 18 combined land use/land cover types (Table 1). Designation of the land use combination categories (CCs) is based on data from the Swiss land use statistics (2004–2009) which are derived from aerial photography interpretation (SFSO 2013) and Table 6-6 of the Swiss National Inventory report (FOEN 2016b). Emission factors and carbon stocks on forest land are derived from the Swiss national forest inventories (FOEN 2016b). Carbon stock/biomass values for CCs outside of forest are based on best available research studies, field surveys and measurements, or alternatively, expert estimates and/or IPCC defaults values where no detailed biomass data is available (FOEN 2016b). Several CCs include no information on tree biomass at all. Cropland (CC21) estimates are calculated as area weighted means for the seven most important annual crops in Switzerland; any tree biomass is not included. Biomass estimates for permanent grasslands (CC31) are on the basis of management type (meadows and pastures (Lowland or Alpine)). Carbon stocks on CCs shrub vegetation (32) and copse (34) are assumed to be equal to that on brush forest which has been estimated in separate research (Düggelin and Abegg 2011) at 20.45 T C/ha (FOEN 2016b). CC33 ‘vineyards, low-stem orchards, tree nurseries’ is defined generally with a 1 m maximum stem height. Here, carbon stock has been estimated based on measurement of area and relevant research on average stand density and biomass values per plant for vineyards and expert opinion for low stem orchards (FOEN 2016b). Estimates of biomass of large fruit trees from relevant research are combined with measures of the mean stand density to obtain carbon stock estimates for CC35 orchards; the carbon stock estimate for permanent grasslands (31) is added to represent the grassy understorey of most orchards (FOEN 2016b). Carbon stocks on stony grassland (CC36) are assumed to be 35% of the levels on brush forest since approximately 35% of the surface of this CC is covered by vegetation. Unproductive grassland (CC37) is assumed to have carbon stocks at the mean value of permanent grassland across all elevation zones. Surface waters (CC41), buildings and constructions (CC51) and other land (CC61) are defined as having no carbon stocks in living biomass (FOEN 2016b). Carbon stocks for unproductive wetland (CC42) and herbaceous biomass, shrubs and trees in settlements (CC52-CC54) were estimated by relevant research (FOEN 2016b).

**Table 1:** Comparison between results for modelled living tree biomass (ALS data) and Swiss GHGI (FOEN 2016b) carbon stocks in living biomass per CC in average tonnes of carbon per hectare over the whole CC area (excluding masked areas); CC1X corresponds to forest land, CC21 to cropland, CC3X to grassland, CC4X to wetlands, CC5X to settlements and CC61 to other land, respectively

Land use CC	GHGI estimate T C/ha	ALS model estimate T C/ha	GHGI estimate includes tree (>3 m) biomass
11 Afforestations	9.08	43.33	Y
12 Productive forest	121.57–128.31	115.28	Y
13 Unproductive forest	31.61	30.82	Y
21 Cropland	4.51–4.93	8.49	N
31 Permanent grassland	7.04	15.09	N
32 Shrub vegetation	20.45	8.66	Y
33 Vineyards, low-stem orchards, tree nurseries	3.74	14.15	N
34 Copse	20.45	45.90	Y
35 Orchards	24.32	29.10	Y
36 Stony grassland	7.16	3.12	Y
37 Unproductive grassland	7.01	6.41	N
41 Surface waters	0	6.13	N
42 Unproductive wetland	6.50	19.60	Y
51 Buildings and constructions	0	31.21	N
52 Herbaceous biomass in settlements	9.54	30.24	Y
53 Shrubs in settlements	15.43	35.57	Y
54 Trees in settlements	20.72	48.51	Y
61 Other land	0	2.92	N

In CCs with annually changing data (productive forest (12) and cropland (21)), the range of average values for the period 2001–2014 (time span of the model input data) is given. Results stratified by elevation and NFI region are available in the supplementary material

### Modelling

Living tree biomass (above ground and below ground in T/ha) is the response variable for all modelling. The Swiss national forest inventory reports tree biomass for each single tree on a plot and extrapolates them to per hectare values. Living tree biomass values were available for all forested areas. Plots at forest and stand edges are excluded from the modelling process to avoid errors when scaling from individual tree measures to per hectare values. This resulted in 5562 plot level measurements for modelling input including data from NFIs 3 and 4, where some plots have measurements from both NFIs 3 and 4, and some from one only.

In order to compare the capabilities of ALS data to that of aerial stereo imagery for modelling tree biomass, explanatory variables included per plot (25 m diameter) metrics from point cloud data from both ALS and ADS (in separate models) as follows: mean height of all points over 3 m in height (avg), a variety of height percentiles (50, 70, 80, 90, 95), standard deviation of height of returns over 3 m (std) and vegetation canopy cover derived from points (cov = number of first returns above 3 m divided by the number of all first returns). All metrics were derived using Lastools lascanopy tool (Isenburg, http://rapidlasso.com/lastools) on normalised data. NDVI was derived from a cloud-free composite of DMC imagery data dated May 2012. Climate variables employed as explanatory variables were as follows: average summer temperature, average summer precipitation, site water balance (estimate of the water available to plants during a water year, based on precipitation, evapotranspiration, soils and topographic position), potential evapotranspiration and global potential short wave radiation.

Model areas were stratified by elevation in three classes representative of the Plateau (0–600 m), Pre-Alpine (600–1200 m) and Alpine (above 1200 m). Stratification by national forest inventory production regions, which are similar to bioregions (the Jura, the Plateau, the Pre-Alps, the Alps and the Southern Alps), was also tested but not found to be meaningful.

A number of modelling approaches were tested including linear least-squares regression, non-linear least squares regression and random forest models. Model performance was compared largely based on not only model efficiency (equivalent to *R* ^2^ in a linear model) (Pinjuv et al. 2006) but also prediction error calculated using a leave-one-out cross-validation algorithm and *Q* value (Holmgren et al. 2003). *Q* value is defined as the ratio between the standard deviation from the cross-validation (prediction error) and the standard deviation from the regression (root mean square error); the closer this value is to 1, the better. Least-squares methods were used to calibrate the model (R functions lm and nls for linear and non-linear models respectively). For linear models, the AIC backward selection criterion was used to discard unnecessary variables. All the models were evaluated using a leave-one out cross-validation algorithm. In contrast to results reported by Fassnacht et al. (2014), in general, across stratification and point cloud metric source (ALS vs ADS), performance was very similar between linear and non-linear (including random forest) models (Table 2). As such, results reported in this paper are those from the linear least-squares regression approach, since this offers the easiest approach to implement and interpret without compromising performance. The ability to predict tree biomass was also compared between models based on ALS point cloud data and those based on ADS point cloud data using the aforementioned criteria. To determine variable importance a set of models with all possible combinations of the explanatory variables were modelled, with Akaike weights calculated for each model. Relative importance of each explanatory variable is then determined as the sum of Akaike weights over all models in the set where the variable of interest occurs (Burnham and Anderson 2002). The Akaike weights represent the relative likelihood of a model (normalised to sum to 1), given the data and the full set of candidate models (Burnham and Anderson 2002).

**Table 2:** Model performance comparison of linear least-squares regression, non-linear least squares regression and random forest models

Model	Model efficiency	*Q* value
ADS height variables all forest types non linear	0.57	1.0027
ADS height variables all forest types linear	0.55	1.0028
ADS height variables all forest types random forest	0.56	1.0202
ALS height variables all forest types non-linear	0.54	1.0026
ALS height variables all forest types linear	0.58	1.0030
ALS height variables all forest types random forest	0.60	1.0062

To evaluate the performance of the model for non-forest areas, we tested the application of the model in areas of predicted (from NFI field data) tree biomass within forest plots which have characteristics that can be considered similar to non-forest trees (e.g. coppice, talus, disperse forests, low canopy cover).

The best performing models were then applied across the entire set (inside and outside forest) of point cloud data to create a nationwide model of tree biomass. Prior to model application, point cloud data was classified for return type using lasclassify in LAStools and masked for areas of buildings (1.8% of total ALS data area) using the Swiss topographic landscape model with a 2 m building buffer. The linear least squares models were applied to the point cloud metrics on a 25 × 25 m grid. Areas of high voltage powerlines and other cables (such as cable cars and ski lifts) were masked from the final output (approx. 1% of total ALS data area) since powerlines were difficult to classify in a consistent manner across all of the ALS datasets and automated classification procedures often classified them as vegetation which could therefore distort biomass values.

For model validation, modelled tree biomass values (T/ha) were compared with above ground biomass data based on measured field data of TOF from the community of Bern urban tree inventory (Gardi et al. 2016). Gardi et al. (2016) report on above ground carbon density and use a conversion fraction of 0.47 to above ground biomass. To approximate total tree biomass (above ground and below ground) we used the roots-to-shoots ratio of 0.26 (Nowak and Crane 2002). The Swiss GHGI also reports carbon density of biomass and uses the conversion fraction of 0.5 (FOEN 2016b). All values were converted back to biomass with the relevant conversion fraction prior to comparison. Biomass values at the plot level (*n* = 173) (t/ha) were compared and evaluated by *R* ^2^ value. Finally, the resultant models were compared with the current biomass estimates from the Swiss GHGI (FOEN 2016b).

## Results

**Fig. 1:**
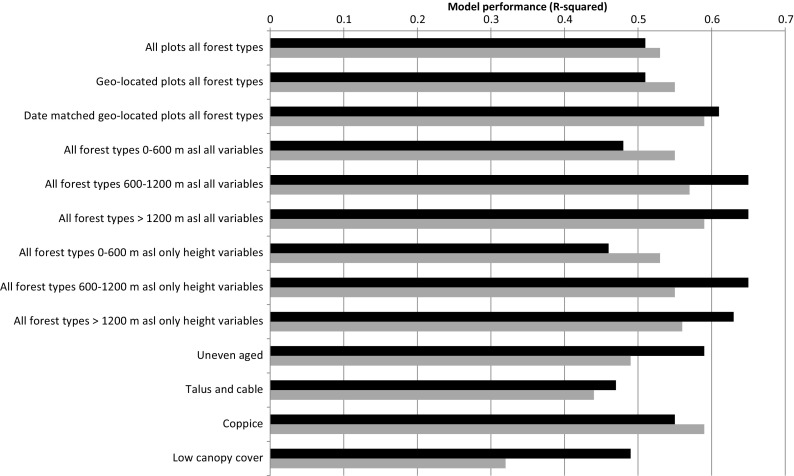
Model performance (*R* ^2^) for models of tree biomass including only ALS (*black*) or ADS (*grey*) point cloud metrics. Importance of stratification by elevation, forest type, date-matching between field data and remotely sensed data and knowledge of exact plot geo-location are examined

Comparison of model performance by *R* ^2^ demonstrated variability depending on the forest type, stratification and the source of point cloud data (Fig. 1). Models built on data from plots with exact geo-locations and where data collection of point-cloud data and field data were within 2 years of each other performed best (*R* ^2^ of 0.60 (ALS) and 0.61 (ADS)). Stratification by elevation improved model performance for models based on ALS data and at elevations over 600 m. The performance of models using ALS data and those using ADS data was generally comparable, with no consistency in which dataset had higher model efficiency. There were larger performance differences in coppice and uneven aged forests (Fig. 1).

**Fig. 2:**
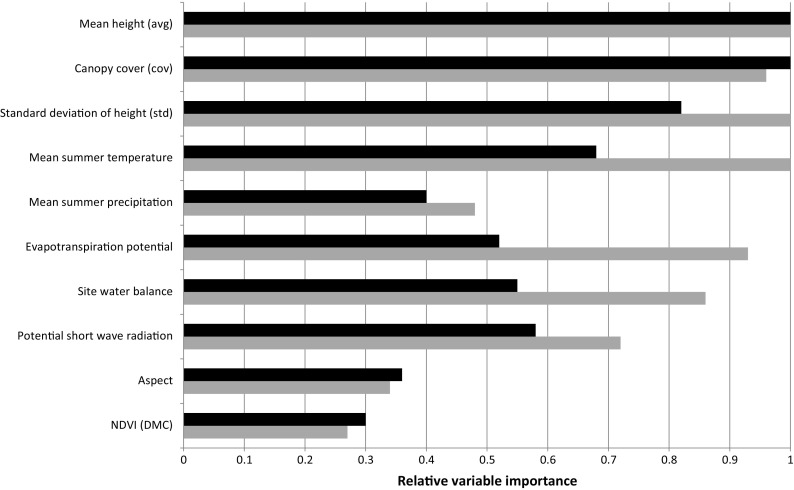
Relative variable importance within the ALS based model (*black*) and the ADS based model (*grey*) calculated through the model averaging approach (Burnham and Anderson 2002)

Evaluation of variable importance by model averaging revealed that height metrics derived from point cloud data were the most important explanatory variables in modelling tree biomass with ALS data, in particular average height within the plot (Fig. 2). However, the relative importance of height variables over other explanatory variables was less pronounced in the ADS model where climatic variables were of similar importance (Fig. 2). Nevertheless, models containing only height metrics performed almost equally as well as those models with climate or NDVI data, suggesting that the benefit of including this data is minor (Fig. 1). Final implementation was the ALS model stratified by elevation and containing height metrics only (Table 3).

**Table 3:** Final implementation of tree biomass model stratified by elevation where avg is mean height of all ALS points over 3 m in height, std is standard deviation of height of returns over 3 m and cov is vegetation canopy cover derived from points as the number of first returns above 3 m divided by the number of all first returns

Strata	Model	Model efficiency	*Q* value	RMSE %
0–600 m asl	−0.641 + 13.732 × avg	0.48	1.0455	39.23
600–1200 m asl	−117.204 + 16.489 × avg – 5.176 × std + 1.494 × cov	0.58	1.0086	37.45
>1200 m asl	−86.325 + 12.827 × avg – 2.449 × std + 2.351 × cov	0.62	1.0542	39.51

All values for 12.5 m radius plot

**Fig. 3:**
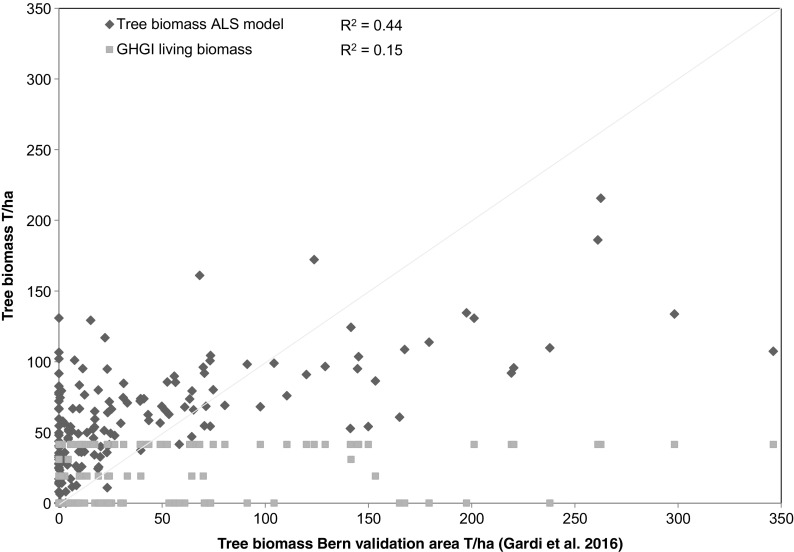
Predictive power of the ALS based model vs the Swiss GHGI biomass estimates for the validation area of the community of Bern, where above ground, carbon density data from the Bern study (Gardi et al. 2016) is converted to AGB using the factor of 0.47 and to total tree biomass (above ground and below ground) using the root-to-shoot ratio of 0.26. The tree biomass models presented in this study offer a far more nuanced model of biomass at 25 m pixel resolution than current nationwide GHGI models (an average value per CC, 1 ha resolution) allowing for greater variability especially in non-forest areas (Fig. 4). The Bern plots were located on areas falling into 4 of the Swiss GHGI CCs: Buildings and Constructions (51), Herbaceous Biomass in Settlements (52), Shrubs in Settlements (53) and Trees in Settlements (54)

Model validation was performed with the ALS based model including only point cloud metrics (Table 3) and showed that within the validation area of the community of Bern urban tree inventory, the performance of our model was relatively low (*R* ^2^ = 0.44), but nevertheless, a large improvement on the estimates of biomass used in reporting to the Swiss GHGI (*R* ^2^ = 0.15) (Fig. 3). However, the GHGI values are assumed average values over each the four CCs in Settlements (1 ha resolution) which do not take into account within CC heterogeneity and as such cannot really be compared to the plot level (25 m diameter) values of the validation data and the ALS model.

In productive and unproductive forest (CC12 and CC13), there is a relatively good agreement between the modelled average value per CC and the GHGI estimate (Table 1). In other CCs, biomass estimates were often considerably different between our model and the Swiss GHGI estimates (Table 1, ALS based model). Within the Bern validation area, while the fit of our model to the field data was not necessarily very good (*R* ^2^ = 0.44), performance is nevertheless a great improvement on that of the GHGI estimates (*R* ^2^ = 0.15; however, note the restrictions mentioned above) with the ALS-based model finding tree biomass under CCs for which the GHGI reports no biomass: surface waters (41), buildings and constructions (51) and other land (61). These categories have no biomass reported in the GHGI largely by virtue of data availability and assumptions within the definition of the CCs, as well as reporting guidelines set out by IPCC (2006) in the case of other land (61). Nevertheless, the finer resolution of the data in the ALS model allows more precise definition of land use type borders, and therefore, accounting for vegetation, for example, at shorelines.

## Discussion and conclusions

This work demonstrates an efficient and easily implementable approach to modelling tree biomass across a large heterogeneous area, relying on either wall-to-wall ALS or spectral imagery datasets. However, the overall performance of the model with model efficiency between 0.48 and 0.62 (Fig. 1, Table 3) (depending on elevation) is relatively low over the heterogeneous area, with high uncertainty with RMSEs in the range of 37.5–39.5%. In addition, due to limitations of training data availability, the accuracy and representativeness of the model may be lower outside of forest areas as demonstrated in the urban validation area. Supplementary data such as climatic data and satellite imagery data resulted in minimal improvements to the models, confirming the importance of vegetation height and structure variables in determining tree biomass (Dubayah and Drake 2000; Hunter et al. 2013; Lefsky et al. 1999) and demonstrating that a simple approach can be effective. We have built on current knowledge on biomass outside forests, e.g. Schnell et al. (2015), and provide a European national model of biomass with a consistent, wall-to-wall approach, both inside and outside of forest.

While stratification by elevation improved model results, stratification by bioregion (NFI production region) did not result in improvements in model performance despite the relatively large and heterogeneous study region of the whole of Switzerland. Stratification by other variables such as forest type (Asner et al. 2010) may also offer improvement in model performance; however, vegetation type and species data is not consistently available for non-forest areas in Switzerland. Only minor improvements in model performance were achieved by including climatic variables in the models. These results suggest that the relationship between vegetation structure and height is relatively stable across Switzerland despite heterogeneous environmental conditions. Similar results were found in tropical forests by Asner and Mascaro (2014) with consistently strong relationships between LiDAR top of canopy height and field measured carbon density across various tropical vegetation types.

It is not uncommon that the exact location of all field plots is not recorded in forest inventories, which rely on theoretical or approximate geo-locations (Johnson et al. 2014). This often implies a trade-off between a lower number of plots with a precise geo-location and a higher number with a larger error in the geo-location of each plot. In this study, the use of plots with exact geo-location offered only slight improvement in model performance for the stereo-imagery based model, suggesting that this factor does not have a significant impact in the case of the Swiss forests. We did find, as would be expected, that matching the dates of field data and remotely sensed data collection as closely as possible offered measureable improvement in model performance. This result emphasises the importance of using regularly updated vegetation structure data and highlights some of the issues in modelling over a large area where field and remote sensing data are very often recorded at different times.

Poor model performance was, in many ways, to be expected in applying models developed with forest data to non-forest areas including agricultural and urban areas, where trees are more varied in structure, form and extent of the crown, species, etc. (McPherson et al. 2013). Application of models representing forest areas, which could be considered as more similar to non-forest areas, such as areas with low canopy cover, did not improve model fit (Fig. 1).

Chave et al. (2004) note that the most important source of error in estimating above ground biomass is related to the choice of allometric function and the underlying estimation methods and sample data. Indeed, it is important to keep in mind that the models we have developed here are predictions of modelled tree biomass data, where biomass is estimated with allometric functions based on simple diameter and height field measurements. There are therefore likely significant errors and biases in the biomass estimates that are our response variable. Uncertainties in the model of course also derive from uncertainties within the input data. This may include misclassification of the point data, for example wires or cables or unmapped buildings. The implications of such issues for final model uncertainties are difficult to determine, but the extent is strongly limited through data masking. Due to the lack of representative field data outside forest areas, spatial estimates of uncertainty are unavailable beyond the overall uncertainties per elevation strata estimated (Table 3).

ALS-derived point cloud data can penetrate the canopy and thus provide structural information between the ground and the canopy surface, unlike point cloud data from aerial imagery which can offer only surface data. Therefore, it is logical to expect that ALS-derived data might result in higher performing models of forest attributes relating to vegetation structure. However, as Pitt et al. (2014) similarly demonstrated for boreal forests, the model using ALS-derived metrics performed only marginally better (Fig. 1) than that using aerial imagery-derived data, and there are distinct advantages in using ADS data for modelling tree biomass within Switzerland. The ADS data is available on a continuous cycle into the foreseeable future allowing repeatable measurements and frequent model revision and improvements, as well as potentially a cost-effective opportunity for monitoring change in tree biomass and related carbon stock (Ginzler and Hobi 2015). However, robust assessments of uncertainties in the static estimates would be needed in order to be able to assess change over time. While the ADS aerial photography is part of a consistent national imagery capture campaign, the ALS dataset is a patchwork of various campaigns, captured for different purposes with significant variations in point density, sensor specifications, time of capture, etc. Each of these factors has implications for the ability to consistently predict tree biomass across a large heterogeneous area.

**Fig. 4:**
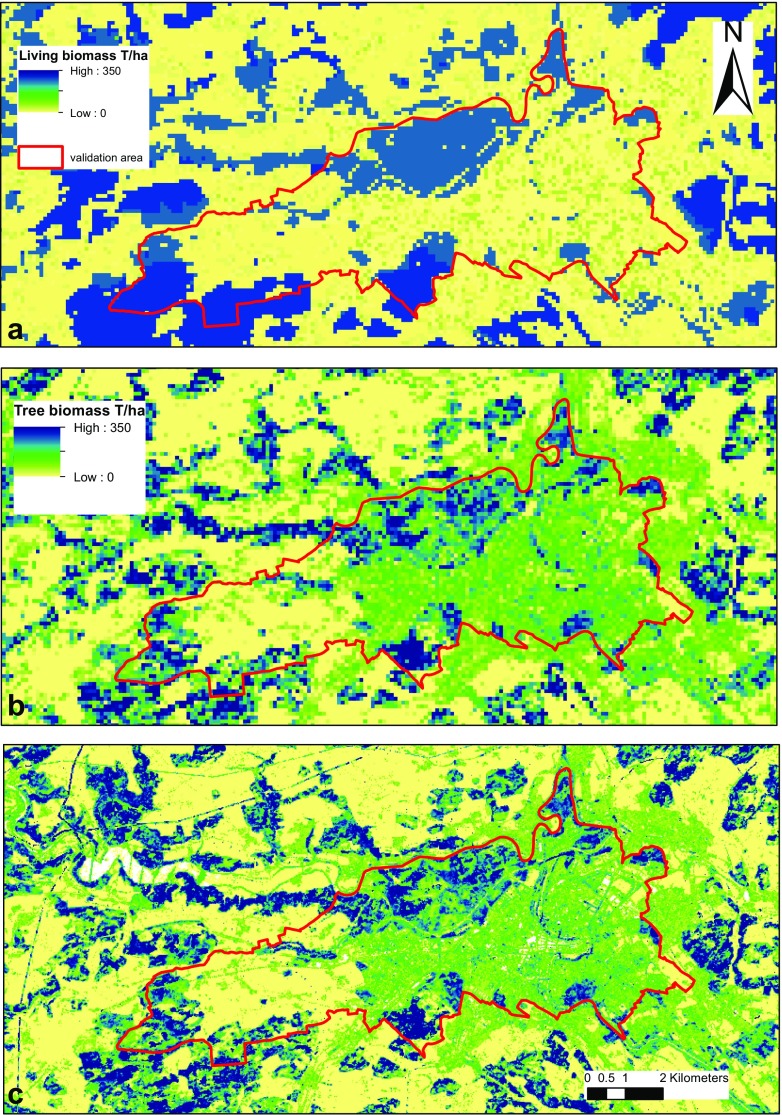
Living tree biomass model estimates for the area of Bern, both inside and outside the validation area, for **a** the current GHGI estimates (FOEN 2016b) per CC mapped spatially, **b** the model resulting from this study using ALS point cloud data resampled to 100 m resolution (same resolution as the GHGI data) and **c** the model resulting from this study using ALS point cloud data at the original 25 m (equivalent of plot size) resolution

Unfortunately, only a small amount of data outside of forests was available to evaluate the models, and the available data was for above ground tree biomass only, while the GHGI and our model were for above and below ground biomass. Indeed, the performance of the models in these evaluation areas was relatively low. In general, the model overestimates biomass for areas with lower biomass and underestimates areas of higher biomass within these non-forest plots. Nevertheless, the models are still a large improvement on the current biomass estimates from the Swiss GHGI (FOEN 2016b) within the validation area, although this was limited to settlement CCs. The ALS model offers the advantage of providing greater spatial resolution in the biomass model and therefore can account for more variability (Fig. 4).

Considerable improvement could be made to the models by acquiring a larger validation dataset, covering a greater area and additional land use types. Currently, the validation dataset covers only a small area of the settlements CCs (CC5X); further validation sites in other parts of the settlement CCs would allow a better understanding of spatial heterogeneity in the predictive ability of the models within those CCs. Validation and calibration data for TOF in other CCs such as grassland (CC3X) and cropland (CC21) would also improve the understanding of the model performance outside of forests. With closer date matching between field data and remotely sensed data, the predictive ability of the models would be improved. The ongoing rolling update of ADS data combined with the continuous national forest inventory program should offer such opportunity. A rolling nationwide ALS campaign with consistency in data and sensor specifications would of course be highly beneficial and allow for improvement in model performance.

The methodology presented aims to model tree biomass and in particular to focus on trees outside forest, which are often otherwise not well represented in nationwide biomass estimates (Schnell et al. 2015) including for Switzerland. The underlying data driving our models comes from NFI data and in the CCs relevant to forest (productive and unproductive forest, CC12 and CC13); the agreement between the modelled average value per CC and the GHGI estimate is good (Table 1). The differences for afforestations (CC11) can be partly attributed to the GHGI methodology for estimating carbon stock on afforestations where only samples dominated by small trees (at least 85% of the trees with a DBH smaller or equal to 20 cm) were included (Thürig and Traub 2015). However, the results show an often large difference in modelled tree biomass from those of the current estimates in the Swiss GHGI for most CCs outside of forest (FOEN 2016b). In interpretation of the results, it is important to note the differences in resolution and type of the different data sources and assumptions made for individual CCs. GHGI CCs are considered homogeneous within the category and are defined from aerial photography interpretation based on a single point per hectare (SFSO 2013). The ALS and ADS metrics, however, were modelled from continuous point cloud data and as representative of vegetation structure for 25 m pixels.

The ALS model also offers the ability to recognise woody biomass on CCs which, due to lack of data availability currently only account for non-woody biomass such as cropland (21) and Permanent and unproductive grassland (31 and 37) and vineyards, low-stem orchards, tree nurseries (CC33) (cf. Table 1). Such quantification of biomass on non-forest areas is becoming increasingly important with the current trend of increasing urbanisation and land use change and the need for ecosystem services assessment. It is particularly relevant in human settlement areas, which have been shown to have the potential to store as much carbon as tropical forests, e.g. in the USA (Churkina et al. 2010). In CC32 shrub vegetation and CC36 stony grassland, where living biomass is predominately made up of (shrub) vegetation below 3 m, which the ALS models do not capture, and tree biomass is included in the GHGI estimate, biomass tends to be underestimated. In addition, biomass estimates for CCs which have a significant coverage in high elevations (such as shrub vegetation CC32, stony grassland CC36 or permanent grassland CC37) may be biased since the ALS coverage does not cover high elevations above approximately 2000 m asl.

The GHGI definitions of certain CCs such as building and constructions (51), surface waters (41) and other land (61) assume no biomass present by (Swiss) definition. The 1 ha grid and point-based classification methodology of the land use data used in the GHGI means that the presence of single trees or patches of trees within an 1 ha area is often not considered, but these can be accounted for with point cloud data in the ALS model. In addition, borders of given land use CCs, such as surface waters (41), are relatively coarsely defined in the GHGI which may result in significant tree vegetation (riparian and shore vegetation) being defined as water and therefore as having no biomass but which would be included in the ALS model as tree biomass.

TOF are currently not or inadequately considered in LULUCF reporting. The modelling approach used in this study provides reasonable biomass estimates for forest and non-forest trees and will largely contribute to the continual improvement of the Swiss GHG inventory. With repeated ALS and or ADS acquisition, particularly if data is captured with similar specifications over time, there is also potential to use this type of model to measure changes in carbon stocks over time which could be particularly valuable for carbon accounting such as within the GHGI. However, careful assessment of model uncertainties would be necessary to be able to estimate change, including estimates of uncertainties in the response variables and the explanatory variables.

## Electronic supplementary material

Table A1 Comparison between results for modelled living tree biomass (ALS data) and Swiss GHGI (FOEN 2016b) carbon stocks in living biomass per CC in average tonnes of carbon per ha; CC1X corresponds to Forest Land, CC21 to Cropland, CC3X to Grassland, CC4X to Wetlands, CC5X to Settlements, and CC61 to Other Land, respectively. In CCs with annually changing data (Productive Forest (12) and Cropland (21)) the range of average values for the period 2001-2014 (time span of the model input data) is given. Results are stratified by elevation and NFI region, except where noted as n.s. (no stratification). Area in ha corresponds to the area of the CC as defined by the 2004-2009 Swiss land use statistics data and the conversion matrix of Table 6-6 of the Swiss National Inventory report (FOEN 2016b), excluding areas masked out for powerlines in this study. (DOCX 27.6 kb)
