# Supplementary material for: Tree biomass in the Swiss landscape: nationwide modelling for improved accounting for forest and non-forest trees
Source: Environ Monit Assess. 2017 Feb 15;189(3):106. doi: 10.1007/s10661-017-5816-7 (PMC5310548; doi:10.1007/s10661-017-5816-7)
Supplement: Supplementary file 1 — Comparison between results for modelled living tree biomass (ALS data) and Swiss GHGI (FOEN 2016b) carbon stocks in living biomass per CC in average tonnes of carbon per ha; CC1X corresponds to Forest Land, CC21 to Cropland, CC3X to Grassland, CC4X to Wetlands, CC5X to Settlements, and CC61 to Other Land, respectively. In CCs with annually changing data (Productive Forest (12) and Cropland (21)) the range of average values for the period 2001-2014 (time span of the model input data) is given. Results are stratified by elevation and NFI region, except where noted as n.s. (no stratification). Area in ha corresponds to the area of the CC as defined by the 2004-2009 Swiss land use statistics data and the conversion matrix of Table 6-6 of the Swiss National Inventory report (FOEN 2016b), excluding areas masked out for powerlines in this study. (DOCX 27.6 kb) [file 10661_2017_5816_MOESM1_ESM.docx]

Table A1: Comparison between results for modelled living tree biomass (ALS data) and Swiss GHGI (FOEN 2016b) carbon stocks in living biomass per CC in average tonnes of carbon per ha; CC1X corresponds to Forest Land, CC21 to Cropland, CC3X to Grassland, CC4X to Wetlands, CC5X to Settlements, and CC61 to Other Land, respectively. In CCs with annually changing data (Productive Forest (12) and Cropland (21)) the range of average values for the period 2001-2014 (time span of the model input data) is given. Results are stratified by elevation and NFI region, except where noted as n.s. (no stratification). Area in ha corresponds to the area of the CC as defined by the 2004-2009 Swiss land use statistics data and the conversion matrix of Table 6-6 of the Swiss National Inventory report (FOEN 2016b), excluding areas masked out for powerlines in this study.

| **Land-use code CC** | **NFI region** | **elevation zone z** | **carbon stock in living biomass GHGI** | **carbon stock in tree biomass (ALS model)** | **Area ha** |
| --- | --- | --- | --- | --- | --- |
|  | **Strata** |  | **[T C/ha]** |  | |
| 11 Afforestations | 1 | 1 | 10.00 | 46.44 | 48 |
|  | 1 | 2 | 10.00 | 43.99 | 82 |
|  | 1 | 3 | 7.50 | 74.89 | 8 |
|  | 2 | 1 | 10.00 | 57.70 | 195 |
|  | 2 | 2 | 10.00 | 43.59 | 79 |
|  | 2 | 3 | 7.50 | 99.93 | 2 |
|  | 3 | 1 | 10.00 | 58.21 | 24 |
|  | 3 | 2 | 10.00 | 53.39 | 83 |
|  | 3 | 3 | 7.50 | 50.85 | 47 |
|  | 4 | 1 | 10.00 | 61.85 | 25 |
|  | 4 | 2 | 10.00 | 34.52 | 51 |
|  | 4 | 3 | 7.50 | 30.35 | 275 |
|  | 5 | 1 | 10.00 | 37.87 | 9 |
|  | 5 | 2 | 10.00 | 16.08 | 3 |
|  | 5 | 3 | 7.50 | 9.08 | 37 |
| 12 Productive forest | 1 | 1 | 127.26 - 129.46 | 107.88 | 53274 |
|  | 1 | 2 | 133.04 - 137.21 | 120.06 | 118884 |
|  | 1 | 3 | 92.07 - 104.77 | 113.20 | 24281 |
|  | 2 | 1 | 130.93 - 134.70 | 125.22 | 130748 |
|  | 2 | 2 | 142.87 - 149.42 | 138.32 | 93545 |
|  | 2 | 3 | 105.90 - 145.66 | 98.19 | 2427 |
|  | 3 | 1 | 152.66 - 162.34 | 127.12 | 9337 |
|  | 3 | 2 | 156.38 - 162.34 | 132.95 | 135999 |
|  | 3 | 3 | 123.64 - 133.70 | 115.25 | 69591 |
|  | 4 | 1 | 106.22 - 122.37 | 110.31 | 8321 |
|  | 4 | 2 | 112.89 - 122.37 | 109.47 | 99056 |
|  | 4 | 3 | 100.71 - 112.35 | 104.02 | 233913 |
|  | 5 | 1 | 83.78 - 95.79 | 94.64 | 18952 |
|  | 5 | 2 | 92.59 - 110.22 | 88.44 | 56980 |
|  | 5 | 3 | 89.41 - 106.17 | 102.55 | 67853 |
| 13 Unproductive forest | 1 | 1 | 38.53 | 63.34 | 114 |
|  | 1 | 2 | 51.10 | 65.54 | 2939 |
|  | 1 | 3 | 51.34 | 62.97 | 2127 |
|  | 2 | 1 | 20.45 | 76.72 | 179 |
|  | 2 | 2 | 35.83 | 80.35 | 160 |
|  | 2 | 3 | 51.33 | 45.58 | 178 |
|  | 3 | 1 | 20.45 | 129.77 | 12 |
|  | 3 | 2 | 47.53 | 64.13 | 1085 |
|  | 3 | 3 | 42.36 | 46.41 | 8474 |
|  | 4 | 1 | 21.60 | 52.05 | 24 |
|  | 4 | 2 | 31.48 | 44.46 | 1393 |
|  | 4 | 3 | 29.88 | 24.71 | 51924 |
|  | 5 | 1 | 20.83 | 48.55 | 245 |
|  | 5 | 2 | 23.82 | 35.76 | 2107 |
|  | 5 | 3 | 24.35 | 26.63 | 19040 |
| 21 Cropland | n.s. | n.s. | 4.51 - 4.93 | 8.49 | 403819 |
| 31 Permanent Grassland | n.s. | 1 | 7.08 | 27.89 | 153884 |
|  | n.s. | 2 | 6.00 | 17.14 | 356121 |
|  | n.s. | 3 | 7.95 | 8.44 | 406463 |
| 32 Shrub Vegetation | n.s. | 1 | 20.45 | 43.95 | 2182 |
|  | n.s. | 2 | 20.45 | 26.85 | 6429 |
|  | n.s. | 3 | 20.45 | 7.28 | 140331 |
| 33 Vineyards et al. | n.s. | n.s. | 3.74 | 14.15 | 25121 |
| 34 Copse | n.s. | 1 | 20.45 | 54.81 | 27100 |
|  | n.s. | 2 | 20.45 | 38.67 | 32776 |
|  | n.s. | 3 | 20.45 | 45.74 | 28729 |
| 35 Orchards | n.s. | n.s. | 24.32 | 29.10 | 952 |
| 36 Stony Grassland | n.s. | n.s. | 7.16 | 3.12 | 149657 |
| 37 Unproductive Grassland | n.s. | n.s. | 7.01 | 6.41 | 64011 |
| 41 Surface Waters | n.s. | n.s. | 0 | 6.13 | 160962 |
| 42 Unproductive Wetland | n.s. | n.s. | 6.50 | 19.60 | 24964 |
| 51 Buildings, Constructions | n.s. | n.s. | 0 | 31.21 | 197489 |
| 52 Herbaceous Biomass in S. | n.s. | n.s. | 9.54 | 30.24 | 79440 |
| 53 Shrubs in Settlements | n.s. | n.s. | 15.43 | 35.57 | 4012 |
| 54 Trees in Settlements | n.s. | n.s. | 20.72 | 48.51 | 24197 |
| 61 Other Land | n.s. | n.s. | 0 | 2.92 | 589530 |
|  |  |  |  |  |  |
| *elevation zones:* | *NFI-regions:* | |  |  |  |
| 1: < 601 m | 1 | Jura |  |  |  |
| 2: 601 - 1200 m | 2 | Central Plateau | |  |  |
| 3: > 1200 m | 3 | Pre-Alps |  |  |  |
|  | 4 | Alps |  |  |  |
|  | 5 | Southern Alps | |  |  |
